# Inkjet Printing of Flexible Transparent Conductive Films with Silver Nanowires Ink

**DOI:** 10.3390/nano11061571

**Published:** 2021-06-15

**Authors:** Xiaoli Wu, Shuyue Wang, Zhengwu Luo, Jiaxin Lu, Kaiwen Lin, Hui Xie, Yuehui Wang, Jing-Ze Li

**Affiliations:** 1 School of Material and Energy, University of Electronic Science and Technology of China, Chengdu 610054, China; 201921030315@std.uestc.edu.cn; 2 Zhongshan Institute, University of Electronic Science and Technology of China, Zhongshan 528402, China; shuyewang125@163.com (S.W.); luozhengwu128@163.com (Z.L.); JIAC13509809967@163.com (J.L.); Xiehuizsedu@126.com (H.X.)

**Keywords:** silver nanowires, conductive ink, inkjet printing, flexible transparent conductive films, photoelectric property

## Abstract

The inkjet printing process is a promising electronic printing technique for large-scale, printed, flexible and stretchable electronics because of features such as its high manufacturing speed, environmental friendliness, simple process, low cost, accurate positioning, and so on. As the base material of printed conductive patterns, conductive ink is the foundation of the development of printed electronics technology, and directly affects the performance and the quality of electronic products. In this paper, conductive ink with silver nanowires (AgNWs) was prepared, with AgNWs of lengths of 2–5 µm and diameters of 20 nm or so, isopropyl alcohol and ethylene glycol as the mixed solvents, and modified polysilane as the wetting agent. We discussed the relationship between the formula of the AgNWs ink and the surface tension, viscosity, contact angle between ink droplet and poly(ethylene) terephthalate (PET) surface, as well as the film-forming properties of the ink. Further, we analyzed the effects of the number of printed layers and the ink concentration of the AgNWs on the microstructures, photoelectric properties and accuracy of the printed patterns, as well as the change in the sheet resistance of the film during different bending cycles. The experimental results show that flexible transparent conductive patterns with a light transmittance of 550 nm of 83.1–88.4% and a sheet resistance of 34.0 Ω∙sq^−1^–78.3 nm∙sq^−1^ can be obtained by using AgNWs ink of 0.38 mg∙mL^−1^ to 0.57 mg∙mL^−1^, a poly (ethylene terephthalate) (PET) substrate temperature of 40 °C, a nozzle temperature of 35 °C, and heat treated at 60 °C for 10 min. These performances indicate the excellent potential of the inkjet printing of AgNWs networks for developing flexible transparent conductive film.

## 1. Introduction

In recent years, flexible electronic devices have attracted the interest of researchers [1,2,3,4,5,6]. To successfully achieve superior flexible electronic devices, it is necessary to investigate the available flexible transparent conductive films (FTCF), one of the functional parts of flexible electronic devices [7,8,9,10,11]. Therefore, attempting to improve the performance of FTCF to optimize the performance of flexible electronic devices has always been a research hotspot [10,11,12,13,14,15,16,17,18]. Over decades, several materials for preparing FTCF have been developed, including graphene [7,8,9,10], carbon nanotubes [11,12,14], silver nanoparticles [13], as well as silver nanowires (AgNWs) [10,15,16,17,18,19,20]. Among them, one-dimensional AgNWs are widely used in FTCF due to characteristics such as a high aspect ratio, large specific surface area, excellent electrical conductivity and the desirable light transmittance of AgNWs networks [15,16,17,18,19,20,21,22,23,24,25,26]. Until now, AgNWs FTCF have been utilized in stretchable devices [21,22], flexible display screens, and wearable sensors [25,26], etc. Meanwhile, many methods have been developed to prepare FTCF, including spray coating [27,28], spin coating [29], gravure printing [30,31,32], screen printing [23,33,34,35], and inkjet printing [36,37,38]. Although these methods have the advantages of their simple operation, high efficiency, time efficiency, and low cost, they still present many challenges [28,29,30,31,32,33], such as the poor uniformity, repeatability and controllability of film microstructures; and the subsequent laser etching process to achieve graphics, which greatly restricts the promotion and application of the AgNWs FTCF in flexible devices. Therefore, it is very necessary to explore the technology of AgNWs FTCF with a high quality, high repeatability, controllability, graphitization, high efficiency, and low cost [21,22,23,24,25,26].

In recent years, inkjet printing electronic technology (IPET) has attracted more and more attention as the most promising printing electronic technology [36,37,38,39,40,41]. IPET is a drop-on-demand, non-contact material jetting process capable of printing circuits of complex geometries directly from a computer-aided design [39,40,41]. In addition to high material utilization rates, low manufacturing costs, and its large-scale manufacturing compared to conventional methods, IPET also has the potential to simplify the manufacturing process of circuits and to provide personalized electronic patterns quickly, thus becoming a new trend in the development of the electronic manufacturing industry. IPET has been successfully applied in the development of various electronic devices such as photovoltaic cells [42], organic thin-film transistors [43], and radio-frequency identification devices [44], etc. It needs to be pointed out that the use of electrohydrodynamically induced fluid flows through fine microcapillary nozzles for the jet printing of patterns significantly improves nozzle clogging and the resolution of patterns [45,46,47,48].

Conductive ink is the pivotal material to realize printing technology and the foundation of the development of printed electronics technology; directly affecting the performance and quality of electronic products [36,37,38,39,40,41]. The components of conductive ink mainly include conductive fillers, solvents, adhesive phases, and functional additives; and the conductivity of a printing pattern is closely related to the type and content of conductive components and additives. High-quality inkjet printing conductive ink needs to meet the following basic conditions [36,37,38,39,40,41,42,43,49,50,51,52]: (1) good stability: that is, the components do not decompose or agglomerate easily; (2) appropriate rheological properties to ensure good printability; (3) good compatibility with the substrate; (4) excellent conductivity after heat treatment; (5) high resolution; (6) low heat treatment temperature. For FTCF, the inkjet printing conductive ink also needs to ensure the good light transmittance of the film, which indicates that silver nanoparticles are not suitable for conductive materials.

So far, a few research groups have carried out research on the preparation of AgNWs conductive film using IPET [36,37,38,39,40,41,42,43,44]. Coleman and his coworkers were the first to report a AgNWs conductive pattern on a PET surface using IPET, displaying sheet resistances as low as 8 Ω·sq^−1^, conductivities as high as 10^5^ S·m^−1^, and a transmittance of ~50% [37]. Hsu and his coworkers fabricated conductive films on a polyimide (PI) substrate using IPET with a mixture of silver nitrate and silver nanowires as ink [49], this was opaque although the film had good conductivity. Huang and his coworkers reported inkjet printing AgNWs networks on a PET substrate with high-concentration AgNWs ink, which was still opaque [38]. There are still many challenges in the fabrication of flexible transparent AgNWs conductive film by IPET, including the appropriate AgNWs and ink formulation, inkjet printing process parameters, heat treatment process, etc. [36,37,38,39,40,41]. In this work, by optimizing AgNWs, solvents, and additives, we prepared AgNWs ink suitable for the inkjet printing process and fabricated patterns on a PET surface using inkjet printing. We discussed the relationship between the formula of the AgNWs ink and the surface tension, viscosity, contact angle between ink droplet and PET surface, as well as the film-forming properties of ink. Further, we analyzed the effects of the number of printed layers and the AgNWs ink concentration on the microstructure, photoelectric properties and pattern accuracy of the printed pattern, as well as the change in the sheet resistance of the film during different bending cycles of outward and inward bending. The experimental results suggest the feasibility and potential application prospects of the IPET in fabricating flexible, transparent, AgNWs conductive films.

## 2. Experimental Section

### 2.1. Materials

A suspension of silver nanowires (AgNWs, IST-NW-S30-ST), with a diameter of ~20 nm and length of 2–5 μm dispersed in ethanol, was purchased from Haitai Naxin Technology (Chengdu) Co., Ltd., Chengdu, China. Isopropyl alcohol (IPA ≥ 99.7%) was purchased from Tianjinshi Baishi Chemical Co., Ltd., Tianjin, China. Ethylene glycol (EG) was purchased from Tianjin Yongda Chemical Reagent Co., Ltd., Tianjin, China. PET as a substrate was purchased from Dinglishen New Materials Co., Ltd., Zhongshan, China. Polyether modified polysiloxane (Silcona 137) as a wetting agent was purchased from Oncell Co., Ltd., Guangzhou, China. All the chemicals were used as received.

### 2.2. Preparation of Silver Nanowires Conductive Ink

The typical preparation process is as follows: an appropriate amount of wetting agent (Polyether modified polysiloxane, Silcona 137) is added to a mixed solution of 15 mL of ethylene glycol and 10 mL of isopropanol. This mixed solution is magnetically stirred at 200 rpm for 5 min, and then sonicated for 15 min to remove air bubbles. Further, 1 mL of 10 mg∙mL^−1^ AgNWs suspension is added to the above mixed solvent and stirred at 200 rpm for 5 min to obtain AgNWs conductive ink with a concentration of 0.38 mg·mL^−1^.

### 2.3. Preparation of the AgNWs Conductive Patterns

**Figure 1:**
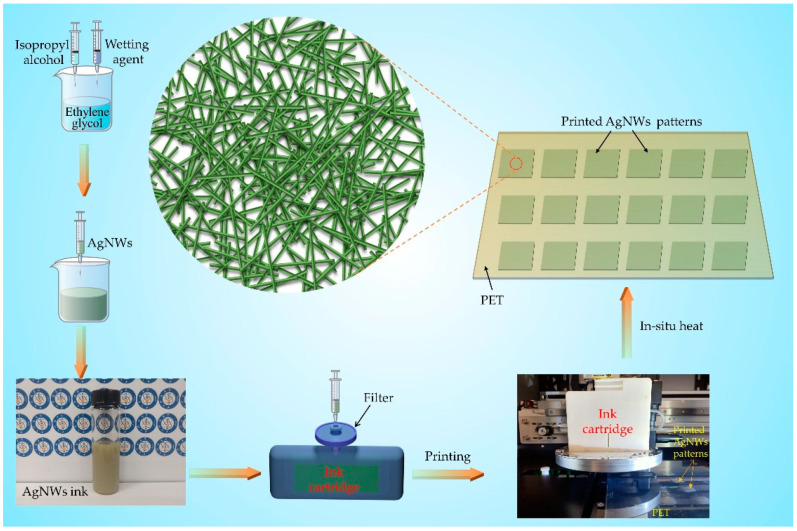
Schematic diagrams of the fabrication process of AgNWs conductive ink, process of inkjet printing, and magnified mode of AgNWs-FTCF in the center.

A microelectronic printer purchased from Shanghai Mifang Electronic Technology Co., Ltd., Shanghai, China—including an ink box with 16 nozzles with diameters of 20 μm arranged in a row—was used to print AgNWs conductive patterns. The number of nozzles used for inkjet printing can be controlled using BitsAssembler (software for controlling microelectronic printer). In addition, the inkjet process of each nozzle is controlled via each piece of piezoelectric ceramics. At first, BitsAssembler was used to design 12 squares with a size of 2 cm × 2 cm that were arranged in an array of 3 rows and 6 columns. The PET substrate was cleaned with deionized water and ethanol successively, and then vacuum-adsorbed on the platform of the inkjet printer. Then the AgNWs ink was injected into the cartridge of the inkjet printer and 1–18 layers, respectively, were printed. The inkjet printing parameters were as follows: voltage of 20 V, 16 nozzles, printing frequency of 7500 Hz, ink droplet spacing of 10 μm. The printing waveform is shown in Figure S1, which was recommended by the equipment manufacturer (see the Support Information for more details). Photographs of the ink cartridge and its operation are shown in Figure S2 and Figure S3, respectively. During the inkjet printing process, the PET substrate was heated to 40 °C and the nozzles were heated to 35 °C, in order to enhance the fluidity of AgNWs ink and facilitate subsequent drying [37]. After printing, the printed patterns were immediately dried on a heater at 80 °C for 10 min. As the solvent evaporated, AgNWs were deposited on the PET substrate. The flexible transparent conductive films with different printed AgNWs layers were finally obtained. Figure 1 shows schematic diagrams of the fabrication process of the AgNWs conductive ink, the process of inkjet printing of patterns, and the magnified mode of the AgNWs-FTCF in the center.

### 2.4. Characterization

A digital viscometer (NDJ-1S, Shanghai Qili Scientific Instrument Co., Ltd., Shanghai, China) was used to measure the viscosity of AgNWs ink. An automatic tension meter (JK99C, Shanghai Zhongchen Digital Technology Equipment Co., Ltd., Shanghai, China) was employed to measure the surface tension of AgNWs ink, and the contact angle measurement (JC2000C1, Shanghai Zhongchen Digital Technology Equipment Co., Ltd., Shanghai, China) was chosen to measure the contact angle of the ink on PET. A sheet resistance meter (DMR-1C, Nanjing Daming Instruments Co., Ltd., Nanjing, China) was utilized to measure the sheet resistance of the flexible transparent conductive film, a haze meter (TH-100, Hangzhou Caipu Technology Co., Ltd., Hangzhou, China) was used to measure the haze value, and a spectrophotometer (UH415 UV, Beijing Techcomp Scientific Instrument Co., Ltd., Beijing, China) was performed to measure the relationship between wavelength and light transmittance.

A scanning electron microscope with a digital camera (SEM, Zeiss sigma 500, Carl Zeiss, Germany), an atomic force microscope (AFM, Dimension Edge, Bruker, Billerica, MA, USA), and an optical microscope (Nikon LV100, Nikon Co., Ltd., Tokyo, Japan) were used to characterize the microstructures of the AgNWs flexible transparent conductive film. An infrared thermal imaging camera (UTI160G, range: −20–350 °C, accuracy: ±2 °C, UNI-T China Co., Ltd., Shenzhen, China) was used to take infrared thermal images. With an infrared lamp (PHILIP PAR38E, 250 W, 0.76–5 μm, Royal Philips Electronics Co., Ltd., Suzhou, China) as the light source, a luminometer (UT382, Uni-Trend Technology (China) Co., Ltd., Dongguan, China) was used to measure the infrared light on the surface of sample. A regulated DC power supply (DPS-3010D, Shenzhen Zhaoxin Electronic Equipment Co., Ltd., Shenzhen, China) was used as the driving power supply.

## 3. Results and Discussion

### 3.1. Properties of AgNWs Inks

It is a challenge to prepare AgNWs FTCF using the inkjet printing process because AgNWs deposit or agglomerate easily, causing nozzle congestion [37,38,39] due to their large size. The optimal size of AgNWs and appropriate rheological properties of AgNWs ink are the keys to ensuring apposite inkjet adaptability [38,40,53,54,55]. Here, we chose a volume ratio of EG:IPA of 1.5:1, as the mixed solvent and wetting agent adjust to the rheological properties of the ink and the contact angle (CA) between the ink droplet and the PET surface. Table 1 presents the viscosity and surface tension of the ink and the CA between the ink droplet and the PET surface at 25 °C, corresponding to varied amounts of wetting agent and an inverse Ohnesorge number. The *Z* value was calculated according to a previously noted formula [53]:

$$Z=\frac{\sqrt{\gamma \rho d}}{\eta }$$

where *η*, *γ*, *ρ* are the viscosity, surface tension, and density of ink, respectively; and *d* is the characteristic length, which generally refers to the diameter of the nozzle or spacing of the droplets. Here, *d* is 20 μm (the diameter of the nozzle).

**Figure 2:**
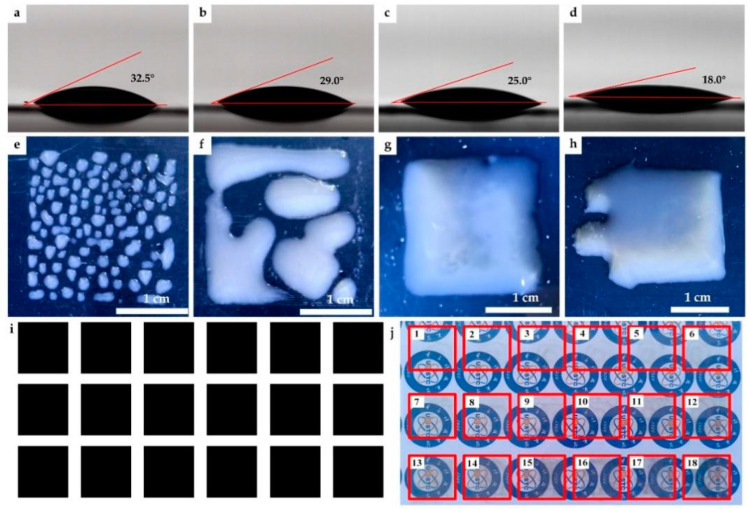
(**a**–**d**) CA images of sample A–D; (**e**–**h**) photographs of the printed patterns without heat treatment with samples of A–D on PET; (**i**) designed square patterns with size of 2 cm × 2 cm by computer; (**j**) photographs of printed patterns with 1–18 layers, respectively, after heat treatment.

Seen from Table 1, it is clear that the viscosity and surface tension of the AgNWs ink without a wetting agent (sample A) are 6.6 mPa·s and 22.869 mN·m^−1^, respectively, and the CA between the ink droplet and the PET surface is 32.5°. The viscosity and surface tension of the AgNWs inks (sample B, sample C, sample D) increase with the increase of the wetting agent, which is related to the characteristics of the wetting agent itself. The Silcona 137 wetting agent is a polyether modified polysiloxane that is generally formed by grafting a copolymerization of polyether and polydimethylsiloxane and its molecular structure contains both hydrophilic polyether segments and hydrophobic polysiloxane segments. It was demonstrated that the *Z* values of the AgNWs inks with the wetting agent were between 3.2 and 2.9, within a suitable range for the inkjet printing [37,53]. Meanwhile, it is important to point out that the microelectronic printer used in our work only has a viscosity requirement, that is, the range of the viscosity of ink is 2–10 mPa∙s to be conducive to the formation of ink droplets. The viscosity requirements of the microelectronic printer are defined by the equipment supplier and determined by the nozzle design of the equipment. As the amount of the wetting agent increases from 5 to 15 μL, the CA between the ink droplet and the PET surface gradually decreases from 32.5° to 18.0°, indicating that the wetting agent (Silcona 137) has good wettability. The CA images of samples A–D are shown in Figure 2a–d.

Samples A–D were used to print patterns of 2 cm × 2 cm on the PET substrate and photographs of the printed patterns before heat treatment are shown in Figure 2e–h. It is clear that each ink droplet of sample A was deposited independently on the PET surface. With the decrease in the CA value, the ink droplets spread to gradually form a continuous liquid film. However, the ink droplets with too small a CA value (18°) easily caused the overflow of the ink droplets. Under our experimental conditions, sample C was the most favorable to obtain the designed pattern, so it was selected for inkjet printing. Square patterns with a size of 2 cm × 2 cm were designed by computer and the above patterns—with 1–18 layers, respectively—were printed on PET substrates. Figure 2i,j show 2 cm × 2 cm patterns designed by computer and the photographs of the printed patterns with 1–18 layers, respectively, after heat treatment. It is obvious that the printed patterns have a regular shape when viewed with the naked eye and with the increase of the number of printed layers, the light transmittance of the films decreases. However, we did not observe satellite droplets or coffee rings in the film, indicating that the distribution of AgNWs on the surface of the PET substrate is uniform, which also indicates that the rheological properties of the AgNWs ink are appropriate. We also discussed the effect of ink droplet spacing on film-forming performance and the experimental results showed that a small ink droplet spacing can cause droplets to easily overflow, while large ink droplet spacing is likely to cause the droplets to fail to connect and blend together (see Supporting Information Figure S4 for more details).

### 3.2. Properties of Ink-Jet Printed Patterns

**Figure 3:**
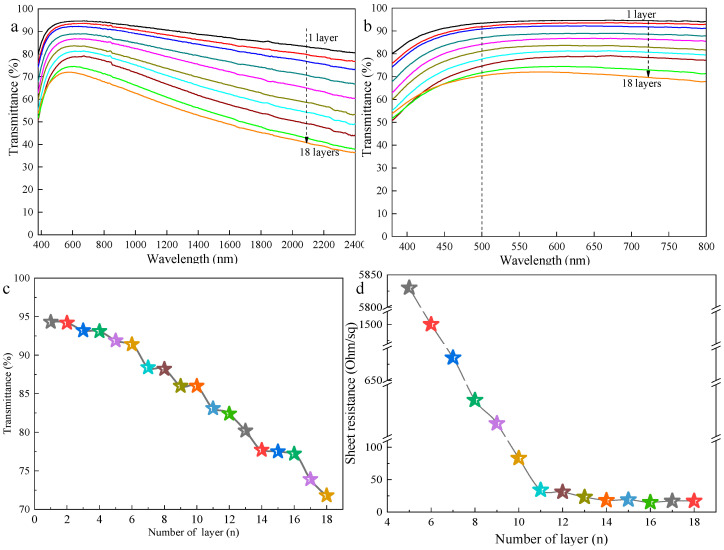
(**a**) Optical transmittance spectra of the samples; (**b**) local magnification of Figure 3a; (**c**) optical transmittance at 550 nm; and (**d**) sheet resistance of samples with different printed layers.

The optical transmittance spectra of the samples with from 1 to 18 printed layers are exhibited in Figure 3a and the local magnification of Figure 3a is shown in Figure 3b. From Figure 3, a gradual decrease in the transmittance of the printed patterns as the number of the printed layer increases can be observed. At 550 nm, the optical transmittance of the films with the printed layers of 2, 4, 6, 8, 10, 12, 14, 16, and 18 were 94.2%, 93.1%, 91.4%, 88.2%, 86.0%, 82.4%, 77.7%, 77.2%, and 71.8%, respectively (Figure 3c). We also measured the sheet resistances of samples with different numbers of printed layers, as shown in Figure 3d. The sheet resistance of the films could not be detected when the number of layers of printed patterns was less than 4. As the number of printed layers increases from 5 to 11, the sheet resistance of the patterns drops almost linearly from 5830 Ω·sq^−1^ to 34 Ω·sq^−1^. After that, with the increase in the number of printed layers, the sheet resistance of the printed patterns slowly decreases until the number of printed layers reaches 16, when the sheet resistance of the printed patterns remains unchanged at about 17 Ω·sq^−1^. As reported before, the deposition density of AgNWs on the PET substrate increases as the number of printed layers increases, leading to a decrease in the light transmittance of the film accompanied by an improvement in the conductivity of the film [27,28,56]. Due to the increase in the amount of AgNWs deposited on the surface of the PET substrate, the dispersed AgNWs gradually stack and overlap each other to form continuous effective conductive networks [7,27].

**Figure 4:**
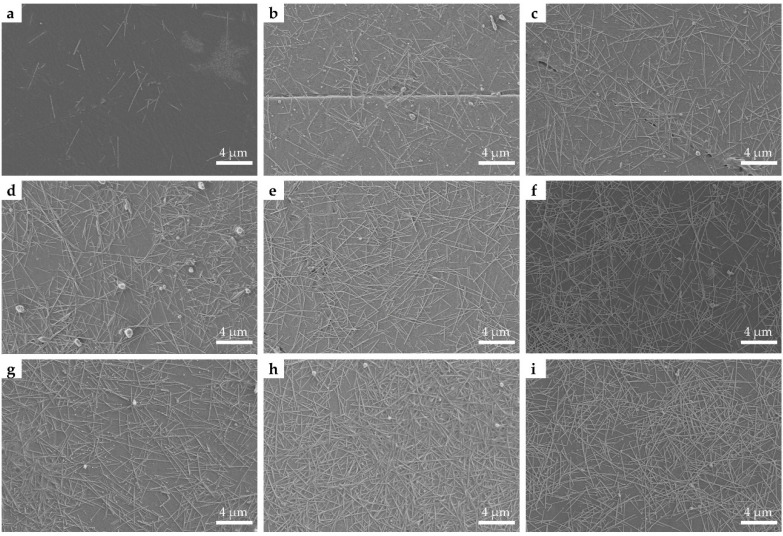
SEM images of printed patterns with the printed layers of 2 (**a**), 4 (**b**), 6 (**c**), 8 (**d**), 10 (**e**), 12 (**f**), 14 (**g**), 16 (**h**), and 18 layers (**i**).

Figure 4 shows SEM images of the printed patterns with 2 (Figure 4a), 4 (Figure 4b), 6 (Figure 4c), 8 (Figure 4d), 10 (Figure 4e), 12 (Figure 4f), 14 (Figure 4g), 16 (Figure 4h), and 18 layers (Figure 4i) printed layers, respectively. It is clear that the AgNWs were randomly distributed on the surface of the PET substrates. There is only a small amount of AgNWs deposited on the PET substrate when the number of printed layers is 2. With the increase in the number of printed layers, the amount of AgNWs deposited on the PET substrate increases gradually. When the number of printed layers is 12, it can be seen that the AgNWs are overlapped together to form connected networks. Previous studies have shown that long AgNWs can enhance the conductivity of AgNWs networks and a high aspect ratio of AgNWs improves the photoelectric performance of AgNWs networks [33,34,48,57]. However, in our experiments, long AgNWs were not suitable for inkjet printing because they can easily lead to nozzle clogging. It should be pointed out that the distribution of AgNWs on the PET substrate is relatively uniform, demonstrating the uniformity of these printed features. Coleman and coworkers reported fabricating AgNWs networks in well-defined patterns by inkjet printing for the first time, obtaining a semi-transparent AgNWs pattern [37]. However, our experimental results demonstrate that the controlled deposition of AgNWs transparent conductive networks in well-defined patterns can be obtained by inkjet printing, with transmittance values at 550 nm ranging from 82.1% to 86.1% and the sheet resistances of the corresponding films ranging from 23 to 86 Ω∙sq^−1^. There was no clogging of the nozzle when we used the self-made AgNWs ink for inkjet printing and the AgNWs ink can be redispersed for inkjet printing after being stored in the fridge for three months.

**Figure 5:**
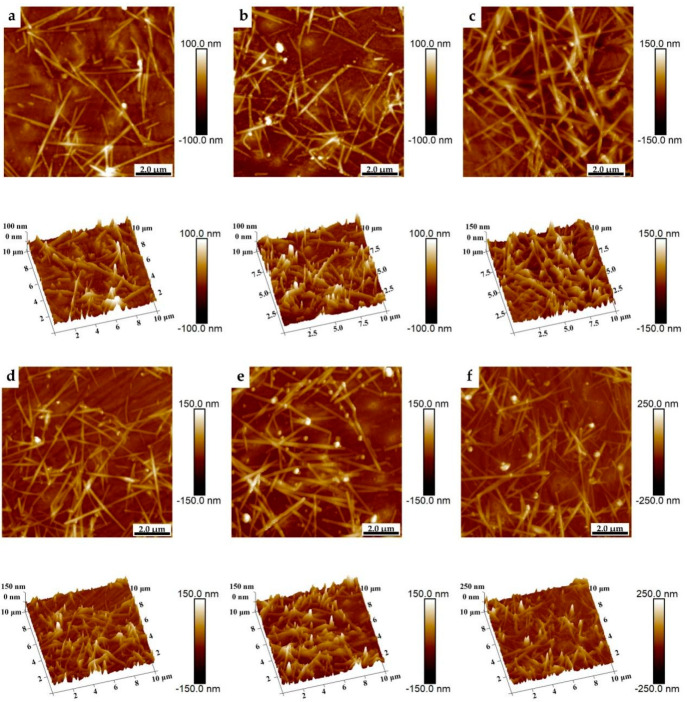
2D and 3D AFM images of the AgNWs patterns with the printed layers of 8 (**a**), 10 (**b**), 12 (**c**), 14 (**d**),16 (**e**), and 18 (**f**).

Figure 5 shows two-dimensional (2D) and three-dimensional (3D) AFM images (operated in tapping mode) of the printed AgNWs patterns with 8 (a), 10 (b), 12 (c), 14 (d), 16 (e), and 18 (f) printed layers, respectively, which reveal the surface topography of the printed AgNWs patterns on the surface of the PET substrates. The measured root mean square (RMS) roughness values of the printed AgNWs patterns with the printed layers of 8, 10, 12, 14, 16, and 18 are 18.3 nm, 21.7 nm, 23.7 nm, 24.2 nm, 30.6 nm, and 35.8 nm, respectively. It is clear that RMS film roughness increases along with the increase in the printed layers. However, the RMS values are less than twice the diameters of the AgNWs, indicating that the printed AgNWs spread out on the PET surface, rather than overlapping and projecting onto it. This is consistent with the phenomenon observed in SEM.

**Figure 6:**
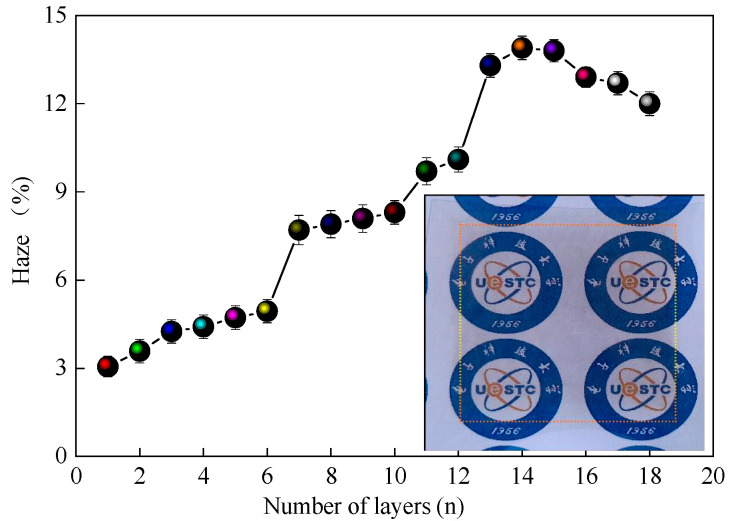
Haze of the printed AgNWs films versus the number of printed layers. The insert shows a 4 × 4 cm^2^ AgNWs film with 16 printed layers on the PET substrate.

As we all know, the optical haze of FTCF is a very important performance parameter of optoelectronic devices, and the haze of FTCF limits the application fields for FTCF. This haze is defined as the ratio of diffuse to specular transmittance and is mainly related to the diameter and distribution uniformity of the AgNWs on the substrate because a thick AgNWs can cause strong light scattering in the visible light range [56]. The AgNWs diameter used in our experiment was about 20 nm, so a single AgNW had a weak scattering of visible light. However, due to the shortness of the AgNWs (5–8 μm in diameter), multiple printed layers were required to obtain good conductivity. The influence of a large number of overlapping and stacking AgNWs on their haze is a problem worthy of our attention. Figure 6 shows the haze of the printed AgNWs patterns versus the number of printed layers. The insert shows a sample of a 4 × 4 cm^2^ AgNWs film with 16 printed layers on the PET substrate. With the increase of the number of printed layers, the haze of the film first increases and then decreases. The haze values of the AgNWs patterns with 1, 3, 6, 9, 12, 15, and 18 printed layers were 3.05%, 4.25%, 4.94%, 8.09%, 10.1%, 13.8%, and 12.0%, respectively, which are significantly higher than we have previously reported [58]. The reasons are mainly related to the size of the AgNW, the distribution of the AgNWs on the substrate, and the thickness of the AgNWs films [10,11,14,56]. It should be pointed out that there are some large silver nanoparticles in the AgNWs suspension used in this work, which also increase the haze of the film. However, following the comprehensive analysis of the above experimental results, it can be seen that the inkjet printing process can still produce a flexible transparent AgNWs conductive film with excellent photoelectric performance and is promising for use in printed conducting applications, although there are still some problems to be solved. We can see from the inserted sample in Figure 6 that the transparency of the film and resolution achievable using this process is excellent.

### 3.3. Influence of Concentration of AgNWs Ink on Photoelectric Properties of Printed Patterns

**Figure 7:**
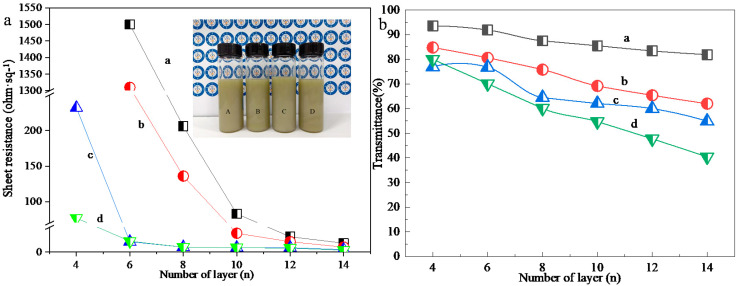
(**a**) Sheet resistances and (**b**) optical transmittance spectra of the samples with concentrations of 0.38 (curve a), 0.57 (curve b), 0.74 (curve c), and 0.91 mg·mL^−1^ (curve d). The insert is a photograph of AgNWs inks with concentrations of 0.38 (A), 0.57 (B), 0.74 (C), and 0.91 mg·mL^−1^ (D).

The concentration of AgNWs ink is not only an important factor affecting the quality of FTCF, but also the rheological properties of the ink and the adaptability of inkjet printing. In order to understand the effects of AgNWs ink concentration on the optoelectronic properties and microstructures of FTCF, we prepared the AgNWs inks with concentrations of 0.38 mg·mL^−1^, 0.57 mg·mL^−1^, 0.74 mg·mL^−1^, and 0.91 mg·mL^−1^ and printed 2 cm × 2 cm patterns with 4, 6, 8, 10, 12, and 14 printed layers on the PET substrate. Figure 7 presents the sheet resistances (Figure 7a) and light transmittance (Figure 7b) of the printed patterns. The insert in Figure 7a contains photographs of the AgNWs inks with concentrations of 0.38 mg·mL^−1^ (A), 0.57 mg·mL^−1^ (B), 0.74 mg·mL^−1^ (C), and 0.91 mg·mL^−1^ (D). It is clear that with the increased concentration of the AgNWs ink, the conductivity of the printed pattern is high and reaches saturation at a low number of printed layers. When the concentrations of AgNWs inks were 0.74 mg·mL^−1^ and 0.91 mg·mL^−1^, the sheet resistances of the 4-layer printing pattern were 232 Ω∙sq^−1^ and 77 Ω∙sq^−1^, which are similar to those of the 8-layer and 10-layer printed patterns with 0.38 mol·L^−1^ of AgNWs ink. The sheet resistances of the 8-layer printing pattern were 10.2 Ω∙sq^−1^ and 10 Ω∙sq^−1^, which are similar to those of the 14-layer printed patterns with 0.38 mol·L^−1^ of AgNWs ink. However, the light transmittance of the printed films with high concentrations AgNWs inks (0.57, 0.74, and 0.91 mg·mL^−1^) decreased significantly. The light transmittances of the 4-layer printed patterns with 0.74 and 0.91 mg·mL^−1^ of AgNWs inks were 80% and 76.9%, which are 14.5% and 17.8% lower than that of the 4-layer printed patterns with 0.38 mol·L^−1^ of AgNWs ink. The light transmittances of the 8-layer printed patterns with 0.74 and 0.91 mol·L^−1^ of AgNWs inks were 64.5% and 64.1%, which are 21.2% and 26.6% lower than that of the 14-layer printed patterns with 0.38 mg·mL^−1^ of AgNWs ink. The photographs of the printed patterns with 0.57, 0.74, and 0.91 mg·mL^−1^ of AgNWs inks are shown in Figure S5 of Supporting Information. The non-uniform distribution and the aggregations of AgNWs on the PET substrates can be observed clearly. The above experimental results show that a high concentration AgNWs ink is not conducive to obtaining high quality FTCF. The reason is that although the concentration of AgNWs ink has little effect on surface tension and CA, in high concentrations of AgNWs ink each droplet contained significant amounts of AgNWs. AgNWs accumulate and aggregate easily under the action of surface tension during the solvent evaporation process, resulting in the non-uniform distribution of the AgNWs and the decrease in light transmittance. In addition, it is noted that the high concentration AgNWs ink did not have good inkjet adaptability due to nozzle clogging. Coleman and coworkers have reported that they obtained translucent AgNWs conductive film on the surface of PET using inkjet printing with the optimal concentration of 0.85 mg·mL^−1^ AgNWs ink [37]. Our experimental results also presented that the light transmittances of printed patterns with high concentrations, are low.

### 3.4. Accuracy of Printed Pattern

**Figure 8:**
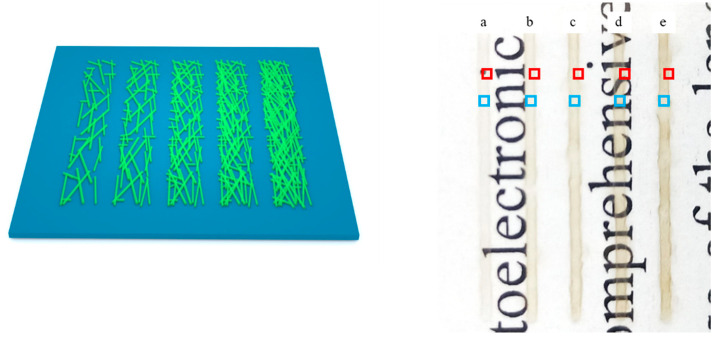
Designed patterns with length of 20 mm and width of 500 μm and different printed layers (**left**), photographs of printed patterns (**right**) with 5 (curve a), 10 (curve b), 15 (curve c), 20 (curve d) and 25 layers (curve e) after heat treatment.

Based on the above research results, we designed (and printed) five linear patterns with lengths of 20 mm and widths of 500 μm and 5, 10, 15, 20 and 25 printed layers, respectively. Figure 8 shows the designed patterns (left in Figure 8), and photographs of the printed patterns (right in Figure 8) with 5 (curve a), 10 (curve b), 15 (curve c), 20 (curve d), and 25 layers (curve e) after heat treatment. As can be seen from Figure 8b, the 15-layer pattern shows the obvious accumulation and overflow of AgNWs at the edges, and with the increase in the number of printed layers, this phenomenon becomes more obvious.

**Figure 9:**
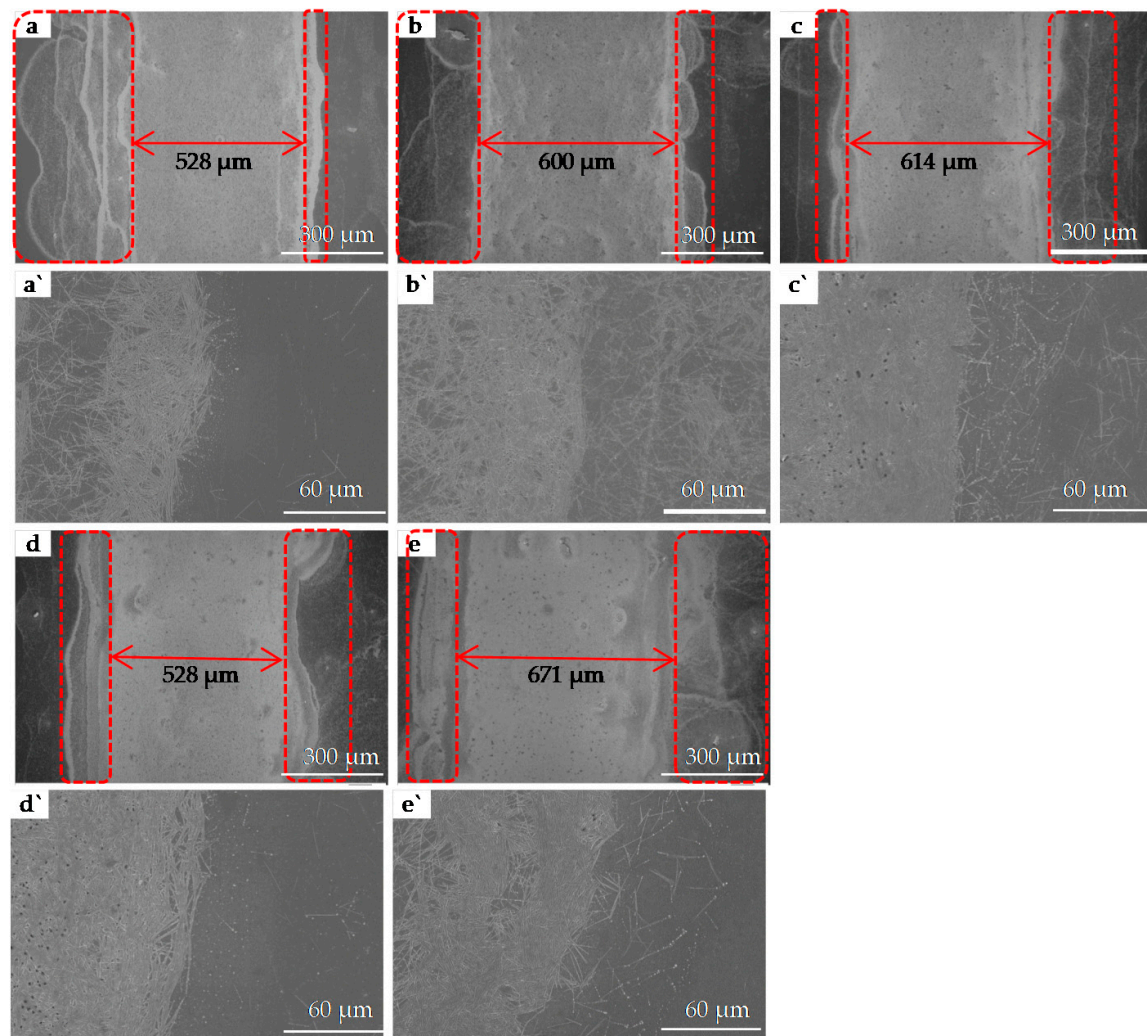
SEM images of the red frame in Figure 8 from the printed patterns (**a)** to (**e**), and (**a`**–**e`**) are the corresponding local magnifications.

**Figure 10:**
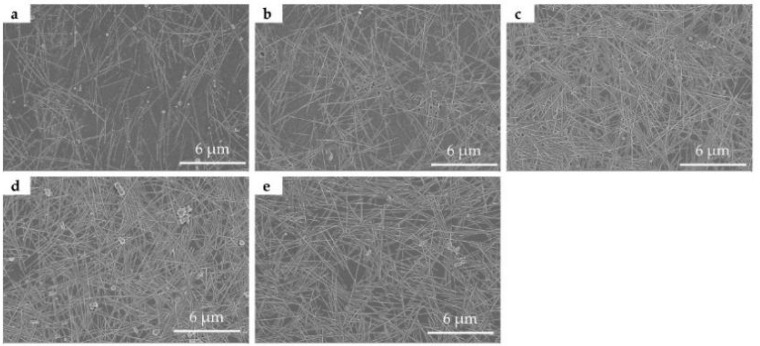
SEM images of the blue frame in Figure 8 from the printed patterns (**a**) to (**e**).

An SEM was used to observe the edge (red frames in Figure 8) and the central (blue frames in Figure 8) portions of the five printed patterns. Figure 9a–e show SEM images of the red frames in Figure 8, from the printed patterns a to e; and Figure 9a`–e` are the corresponding local magnifications. Figure 10 shows SEM images of the blue frame in Figure 8 from the printed patterns (a) to (e). Seen from Figure 9, the edges of the patterns are very irregular and a line-edge roughness of 100–200 μm is observed, which is probably due to the capillary wicking and pinning effect of the AgNWs ink [37]. With the increase in the number of printed layers, the overflow of the AgNWs is obvious on both sides of the pattern. Taking the removed overflow section as the real width (as shown in Figure 9), the real widths of the patterns on the PET substrate with 5, 10, 15, 20, and 25 layers are 528 µm, 600 µm, 614 µm, 528 µm, and 671 µm, respectively. However, note that the distribution of the AgNWs in the middle area of the patterns (the blue frame in Figure 8 from the printed patterns (a) to (e)) is still very uniform.

**Figure 11:**
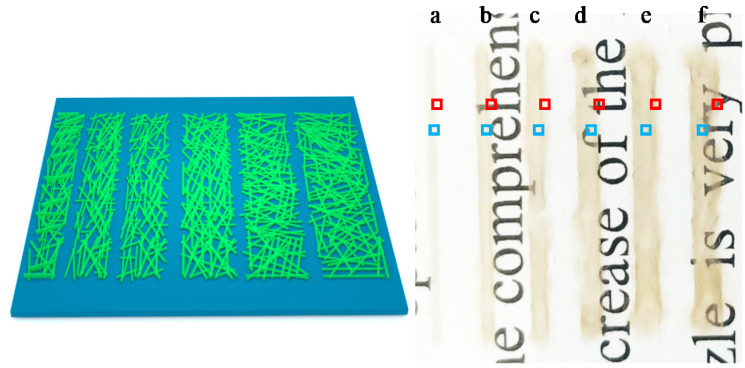
Designed patterns with lengths of 20, and widths of 500 μm and different printed layers (**left**), photographs of printed patterns (**right**) with 200 (curve a), 500 (curve b), 800 (curve c), 1100 (curve d), 1400 (curve e), and 1700 μm (curve f) after heat treatment.

Further, we designed and printed six linear patterns with lengths of 20 mm and widths of 200 μm, 500 μm, 800 μm, 1100 μm, 1400 μm, and 1700 μm, respectively, and each with 20 printed layers. Figure 11 shows the designed patterns (left in Figure 11), and photographs of the printed patterns (right in Figure 11) with 200 (curve a), 500 (curve b), 800 (curve c), 1100 (curve d), 1400 (curve e), and 1700 μm (curve f) after heat treatment. It is obvious that the AgNWs accumulated and overflowed.

**Figure 12:**
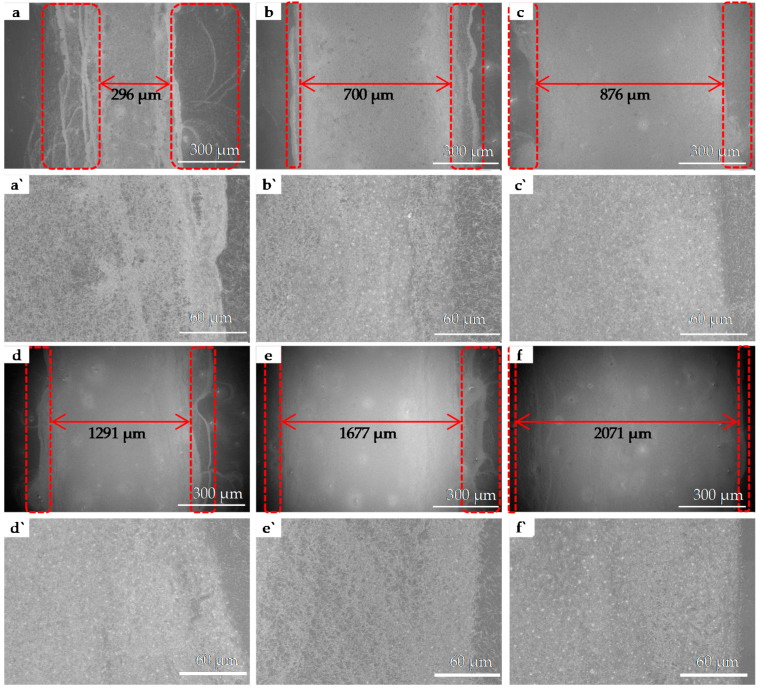
SEM images of the red frame in Figure 11 from the printed pattern (**a**) to (**f**), and (**a`**)–(**f`**) are the corresponding local magnification.

Figure 12a–f show SEM images of the red frames in Figure 11 from the printed patterns a to f, and Figure 11a`–f` are the corresponding local magnifications. The real widths of the patterns on the PET substrate with 200 μm, 500 μm, 800 μm, 1100 μm, 1400 μm, and 1700 μm are 296 μm, 700 μm, 876 μm, 1291 μm, 1577 μm, 1958 µm; and the difference between the real value and the design value is 96 μm, 200 μm, 76 μm, 191 μm, 177 μm, and 258 µm, respectively. Comparing Figure 2j (pattern with 2 × 2 cm^2^), Figure 6 (pattern with 4 × 4 cm^2^), Figure 9 (patterns with 20 mm × 500 μm) and Figure 11 (patterns with 20 mm × (200~1700) μm), we can see that the inkjet-printed narrow linear pattern with AgNWs ink is prone to the phenomenon of AgNWs accumulation and overflow at the edge. The reasons for this are probably related to the large CA between the AgNWs ink droplet and the PET substrate, and the ink drop size.

As described above, it is hard to obtain high-accuracy inkjet printing flexible transparent conductive patterns based on current formulations and processes. Future work is required to further optimize the formula, printing parameters, and heat treatment, etc. Figure 10a–e show SEM images of the blue frames in Figure 8 from the printed patterns a–e. Obviously, the AgNWs are uniform and overlapping each other to form AgNWs networks, indicating the inkjet printing is promising for use in printed conductive applications.

### 3.5. Applications of Inkjet Printting Patterns

**Figure 13:**
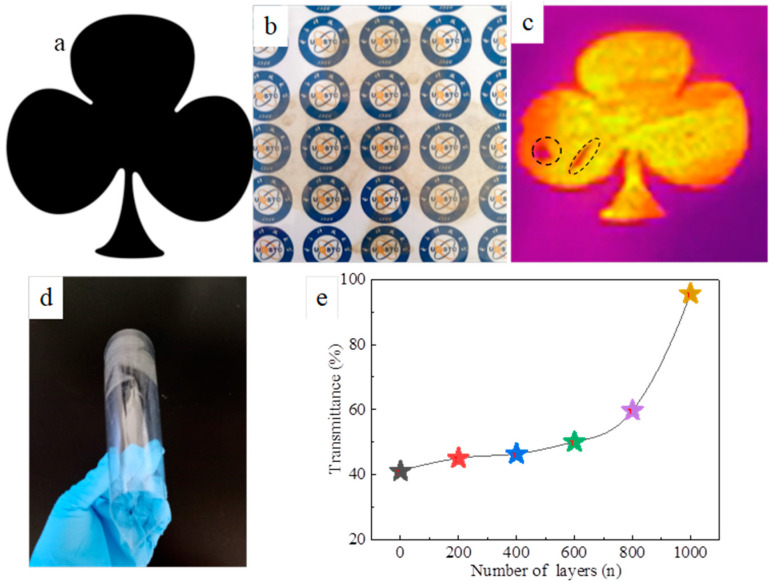
Designed pattern (**a**), printed pattern (**b**), infrared thermal imaging (**c**), film bending photograph (**d**), and the sheet resistance of the film in different bending cycles of outward bending (**e**).

We designed a 4 cm × 4 cm flower pattern to print on the surface of the PET using AgNWs ink. After drying, the film was bent at 200, 400, 600, 800 and 1000 bending cycles of outward and inward bending to test the sheet resistance of the film. Figure 13 shows the designed pattern (Figure 13a), printed pattern (Figure 13b), infrared thermal imaging (Figure 13c), film bending photograph (Figure 13d), and the sheet resistance of the film during different bending cycles of outward and inward bending (Figure 13e). The printed flower-patterned film in Figure 10b is an 11-layer AgNWs structure, with a square resistance of about 40.1 Ω·sq^−1^ and a transmittance of about 84.1%. The infrared thermal imaging shows a uniform heat distribution across the whole surface of the film, indicating that the distribution of the AgNWs on the surface of the PET is generally uniform, except for two obvious defective areas (black dotted circles), which may be related to the droplets from the nozzle. It can be seen from Figure 10e that the relative change in the sheet resistance of the film after 1000 bending cycles of outward and inward bending was less than 2.5, indicating that the mechanical stability of the inkjet-printed AgNWs film is insufficient owing to weak adhesion between the AgNWs and the substrate. However, the film still exhibited a good conductivity and heating performance, as shown in Figure 13c,e.

**Figure 14:**
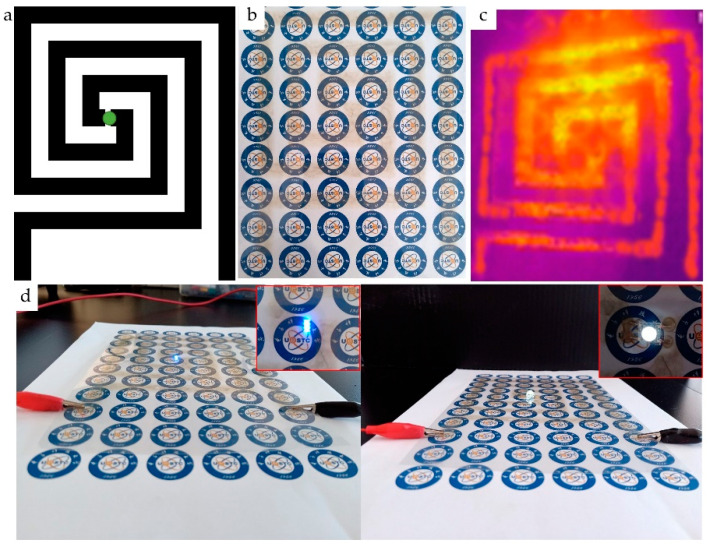
Designed pattern (**a**), printed pattern (**b**), infrared thermal imaging (**c**), and the assembled circuit (**d**), the inserts are local magnifications of (**d**).

We also designed a 4 cm × 4 cm cross finger circuit pattern to print on the surface of the PET using AgNWs ink. After drying, the cross finger circuit pattern and the light emitted diode (LED) bead were assembled into a circuit. Figure 14 shows the designed pattern (Figure 14a), printed pattern (Figure 14b), infrared thermal imaging (Figure 14c), and the assembled circuit (Figure 14d), the inserts are local magnifications of Figure 14d. Seen from Figure 14, the LED’s light worked well, indicating that the printed pattern has good conductivity.

## 4. Conclusions

In summary, inkjet printing silver nanowires (AgNWs) conductive ink was prepared with AgNWs of the length of 2–5 µm and diameter of 20 nm or so as conductive fillers, isopropyl alcohol and ethylene glycol as the mixed solvent, and modified polysilane as the wetting agent. The relationship between the surface tension, viscosity, contact angle between ink droplet and PET surface, and the wetting agent was discussed. The experimental results show that the AgNWs ink with the viscosity of 7–8 mP∙s, surface tension of 23–24 N∙m^−1^ and contact angle (CA) between ink droplet and PET substrate surface of 24°–25° has good inkjet adaptability. The transparent conductive pattern with a light transmittance at 550 nm of 83.1–88.4% and a sheet resistance of 34.0 Ω∙sq^−1^–78.3 nm ∙sq^−1^ can be obtained when the patterns are printed with AgNWs inks of 0.38 mg∙mL^−1^ to 0.57 mg∙mL^−1^ at 40 °C of the poly(ethylene terephthalate) (PET) substrate, a nozzle of 35 °C, and heat-treated at 60 °C for 10 min, 16 holes, and a printing frequency of 7500 Hz. The accuracy of the printed patterns was studied using printed lines with different widths and layers. The experimental results show that the overflow of AgNWs at the two edges decreases the accuracy of the line, and the overflow of AgNWs at the edge decreases with the increase of the width of the line. Therefore, it is required to further optimize the formula, printing parameters, and heat treatment, etc. However, we believe this work is meaningful and interesting to promote the printing of highly conductive, transparent, patterned networks of AgNWs using the inkjet process.

## Figures and Tables

**Table 1:** Composition and performance of AgNWs inks.

Sample	AgNWs (mL)	EG (mL)	IPA (mL)	Wetting Agent (μL)	Viscosity (mPa·s)	Surface Tension (mN·m^−1^)	Contact Angle (°)	Density (g·mL^−1^)	*Z*
A	1	15	10	0	7.6	22.869	32.5	1.0	3.2
B	1	15	10	5	7.0	23.784	29.0	1.0	3.1
C	1	15	10	10	7.1	23.935	25.0	1.0	3.1
D	1	15	10	15	7.7	24.116	18.0	1.0	2.9

Note: Viscosity, density and CA of samples were obtained at 25 °C.

## Supplementary Materials

The following are available online at https://www.mdpi.com/article/10.3390/nano11061571/s1, Figure S1: Jetting waveform parameters and voltage, Figure S2: Photograph of ink box, Figure S3: Photograph of ink box at work, Figure S4: Photographs of the printed patterns without heat treatment with ink droplets spacing of 5 (a), 15 (b), and 20 μm (c), respectively, Figure S5: Photographs of the printed patterns with 0.57 (a), 0.74 (b), and 0.91 mg·mL−1 (c) of AgNWs inks, respectively.
