# Supplementary material for: Inkjet Printing of Flexible Transparent Conductive Films with Silver Nanowires Ink
Source: Nanomaterials (Basel). 2021 Jun 15;11(6):1571. doi: 10.3390/nano11061571 (PMC8232118; doi:10.3390/nano11061571)
Supplement: Supplementary file 1 [file nanomaterials-11-01571-s001.zip › nanomaterials-1235586-supplementary.pdf]

Inkjet Printing of Flexible Transparent Conductive Films with Silver Nanowires Ink

Xiaoli Wu, Shuyue Wang, Zhengwu Luo, Jiaxin Lu, Kaiwen Lin, Hui Xie, Yuehui Wang and Jing-Ze Li

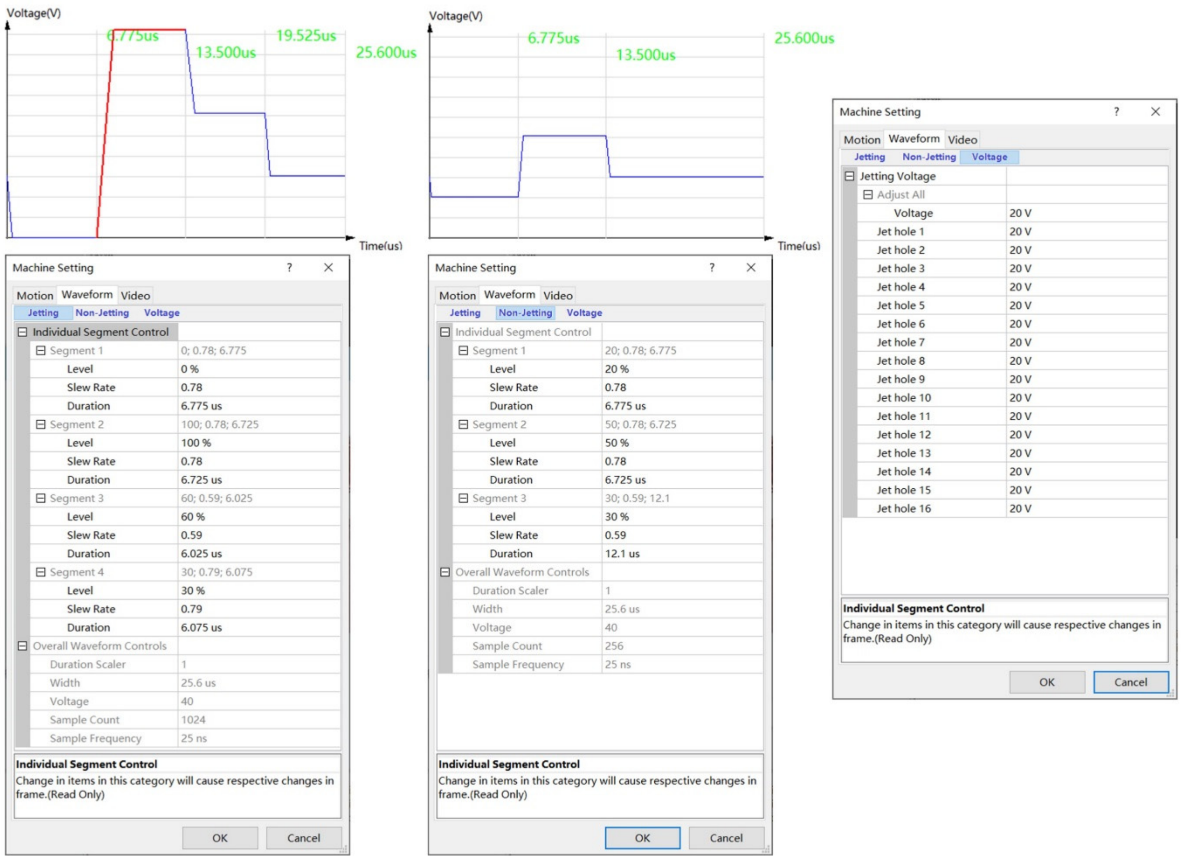

Figure S1. Jetting waveform parameters and voltage.

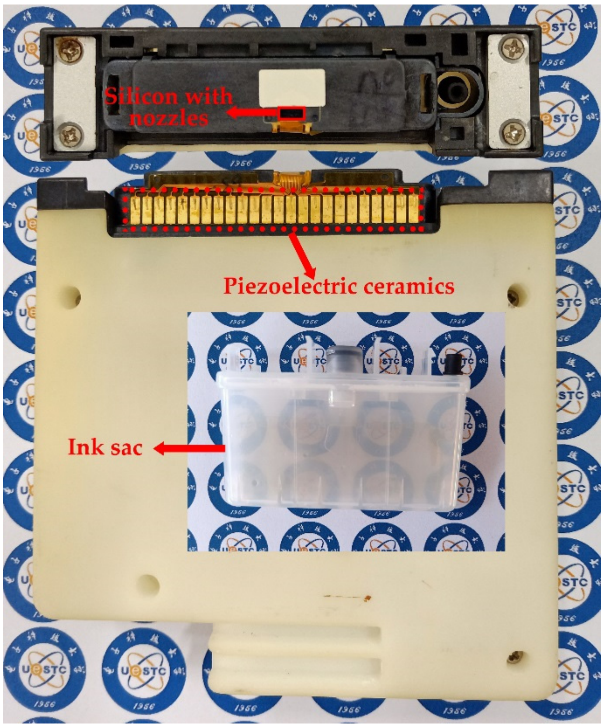

Figure S2. Photograph of ink box.

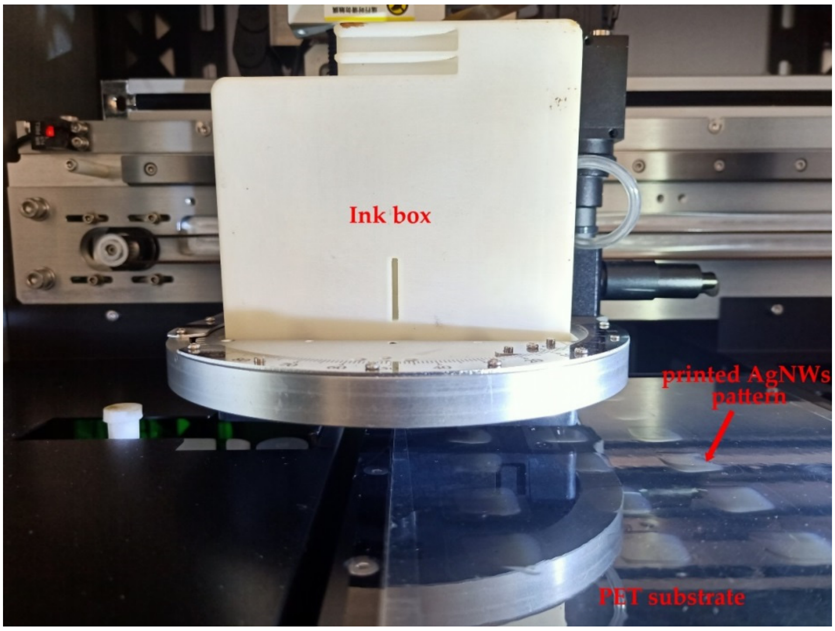

Figure S3. Photograph of ink box at work.

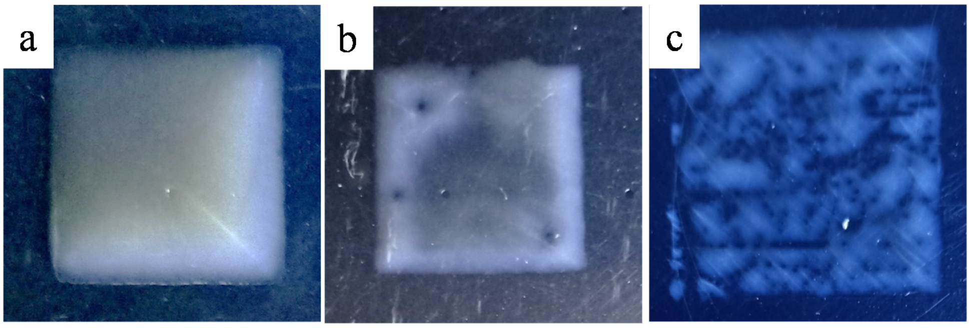

Figure S4. Photographs of the printed patterns without heat treatment with ink droplets spacing of 5 (**a**) 15 (**b**) and 20 µm (**c**), respectively.

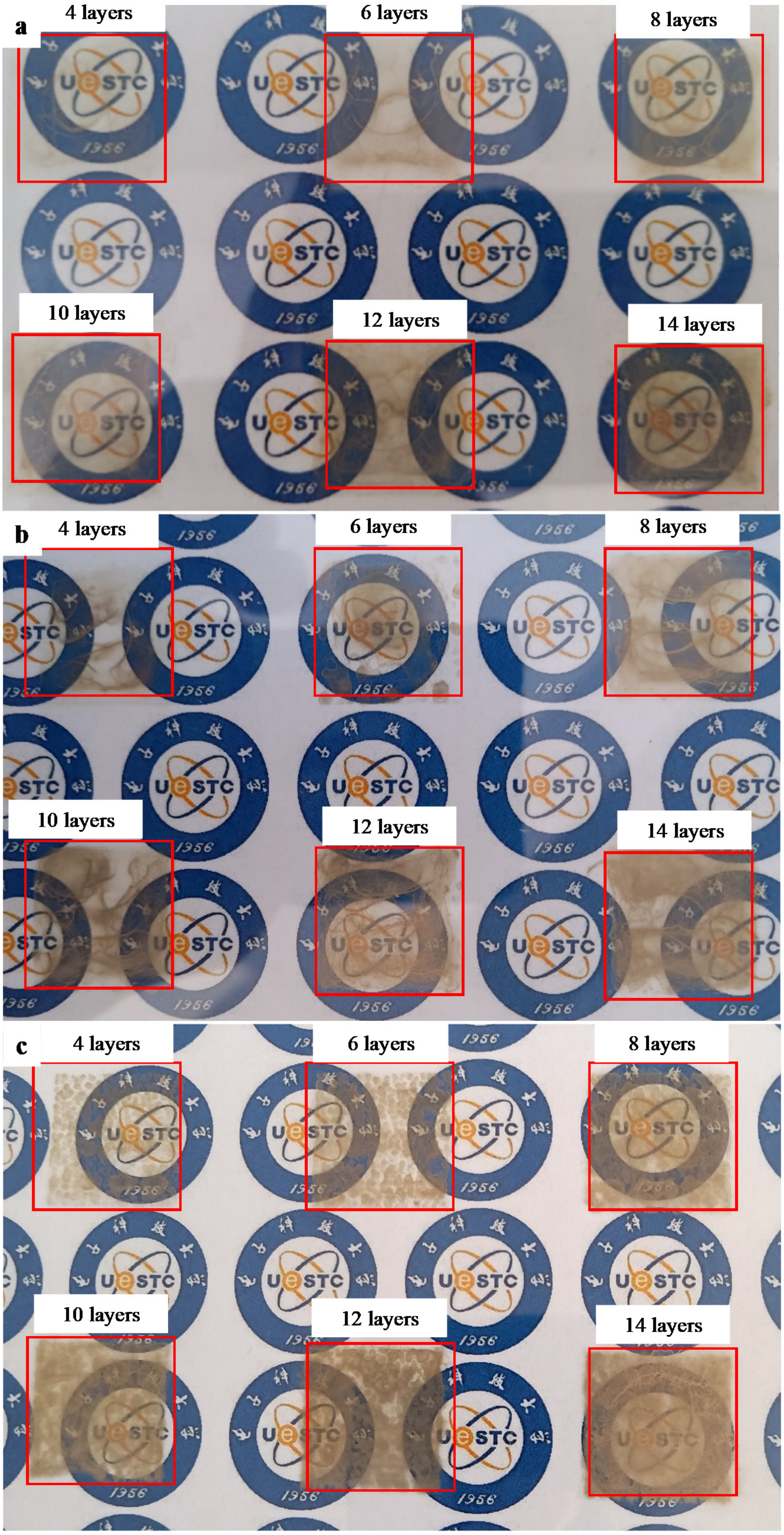

Figure S5 Photographs of the printed patterns with 0.57 (**a**), 0.74 (**b**), and 0.91 mg·mL^{-1} (**c**) of AgNWs inks, respectively.
